# Sustainable rewilding of urban spaces increases microbial diversity

**DOI:** 10.1093/sumbio/qvag020

**Published:** 2026-05-22

**Authors:** Mika Saarenpää, Juulia Manninen, Damiano Cerrone, Juho Rajaniemi, Marja I Roslund, Aki Sinkkonen

**Affiliations:** Ecosystems and Environment Research Programme, Faculty of Biological and Environmental Sciences, University of Helsinki, Niemenkatu 73, 15140 Lahti, Finland; Natural Resources Institute Finland, Horticulute technologies, production systems unit, Latokartanonkaari 9, 00790, Helsinki, Finland; Ecosystems and Environment Research Programme, Faculty of Biological and Environmental Sciences, University of Helsinki, Niemenkatu 73, 15140 Lahti, Finland; Natural Resources Institute Finland, Horticulute technologies, production systems unit, Latokartanonkaari 9, 00790, Helsinki, Finland; Architecture, Faculty of Built Environment, Tampere University, Korkeakoulunkatu 5, 33720 Tampere, Finland; Architecture, Faculty of Built Environment, Tampere University, Korkeakoulunkatu 5, 33720 Tampere, Finland; Natural Resources Institute Finland, Horticulute technologies, production systems unit, Latokartanonkaari 9, 00790, Helsinki, Finland; Natural Resources Institute Finland, Horticulute technologies, production systems unit, Latokartanonkaari 9, 00790, Helsinki, Finland; Department of Environmental and Biological Sciences, Faculty of Science, Forestry and Technology, University of Eastern Finland, Yliopistokatu 2, 80101, Joensuu, Finland

**Keywords:** urban green space, urban greening, urban rewilding, one health, nature-based solutions, microbiota

## Abstract

Soil sealing and biodiversity loss are major drivers of altered microbial communities in urban environments. Little is known about how rewilding reshapes these communities and enriches surrounding sealed surfaces with microbiota. To fill this gap, we first tested whether existing urban green spaces are associated with increased microbial diversity and abundance beyond their boundaries on adjacent impermeable surfaces. We then rewilded a barren, sealed city square using vegetation, compost-based growing medium, and decaying wood. We hypothesized that proximity to green spaces predicts microbial communities more than geographic location, and that rewilding enriches bacterial diversity on nearby sealed surfaces, with diminishing effects across distance. Microbial samples were collected from five green spaces at 0–100 m distances, and from the rewilded and a neighboring non-rewilded square before and after rewilding. In the green space experiment, bacterial richness and relative abundance of *Rhizobacter* declined steadily with distance from green spaces. In the rewilding experiment, bacterial alpha diversity increased compared to baseline conditions and co-occurrence networks contained more nodes and connections post-rewilding. These findings demonstrate that existing green spaces are associated with elevated microbial diversity on surrounding sealed surfaces, and that rewilding urban areas provides a low-cost, nature-based strategy to increase urban microbial diversity.

**Sustainability statement**

This study contributes to SDG 15 (Life on Land) by demonstrating how rewilding urban spaces with vegetation, compost, and decaying wood can increase microbial biodiversity, a critical foundation for healthy terrestrial ecosystems. By enhancing soil fertility, supporting biodiversity conservation, and promoting ecological resilience, the intervention strengthens ecosystem services essential for long-term sustainability. Moreover, increased microbial exposure benefits human immune function and advances the One Health framework, which links human, animal, and environmental well-being. As a cost-effective, scalable approach, rewilding fosters healthier cities and sustainable urban living.

## Introduction

Soil microbiomes provide fundamental ecosystem services. These services include nutrient cycling, soil fertility, water regulation, and climate moderation, all of which are essential for sustainable urban development (Ananyeva et al. 2021). Beyond local benefits, healthy soil microbiomes contribute to the broader framework of planetary health, which recognizes that human well-being is inseparably linked to the integrity of natural systems (Horton et al. 2014). Protecting and restoring urban soil microbial communities is therefore vital for resilient cities and for sustaining ecological processes that support One Health. These microbial benefits are, however, often compromised in urban settings. According to the European Environment Agency (2024), impervious surfaces cover approximately half of the urban areas in the European Union. By 2050, 70% of the world’s population is predicted to live in cities (United Nations, Department of Economic and Social Affairs, Population Division 2019), and as a result, the paved land cover is expected to vastly expand in the following decades (Seto et al. 2012). Paved, impermeable environments disrupt soil processes and sustain less diverse and abundant microbial communities than vegetated habitats (Mhuireach et al. 2016, Puhakka et al. 2019). In general, urbanization homogenizes the microbiota of urban green space soils and favors human pathogens (Delgado-Baquerizo et al. 2021). Since public urban spaces and even private yards have plenty of impervious surfaces (so-called ‘‘grey space’’), there is an unmet need to study how introducing and integrating biodiversity and natural ecosystems into cities affects urban microbial communities. Urban environments often lack key natural elements such as decaying wood, diverse plant cover, and other habitats that sustain soil microbial communities and invertebrates. Efforts to rewild cities have shown promise. For example, revegetated green spaces, like open woodlands, can enhance soil bacterial and fungal communities (Mills et al. 2020). Diverse substrates, such as compost-based growing medium and decaying wood, provide resources for saprotrophs and other decomposers, particularly fungi and bacteria (Johnston et al. 2016, Mieszkin et al. 2021, Embacher et al. 2023), and varied arrays of flowering plants not only support pollinators and other invertebrates but are also associated with greater soil bacterial diversity (Baruch et al. 2021).

The lack of nature in urban areas is also associated with human health. According to the biodiversity hypothesis of health, the loss of natural environments leads to decreased human exposure to environmental microbiota, which in turn contributes to microbial imbalance in the human body and disturbed immune response (Hertzen von et al. 2011, Hanski et al. 2012, Haahtela et al. 2013, Ruokolainen et al. 2017, Haahtela 2019, Mills et al. 2019, 2021). Recurring contact with nature and its microbes can be hard to achieve in cities. Urbanization has been found to reduce exposure to environmental microbes (Parajuli et al. 2018, Gupta et al. 2020, Shan et al. 2020), and urban dwellers have been observed to have distinct microbiota compositions compared to people living in rural areas (Hanski et al. 2012, Ruokolainen et al. 2015, Lehtimäki et al. 2017, 2018). Exposure to diverse environmental microbiota through nature and especially soil is shown to be essential for a balanced human microbiota and normal development of the immune system (Strachan 1989, Rook et al. 2004, Nurminen et al. 2018, Grönroos et al. 2019, Ottman et al. 2019, Roslund et al. 2020, 2021, 2022, Saarenpää et al. 2024). Green space visits have been associated with reduced use of psychotropic, antihypertensive, and asthma medication (Turunen et al. 2023), lower cardiovascular risk through reduced stress (Lanki et al. 2017), and improved child well-being through play and physical activity (Puhakka et al. 2019). Biodiverse vegetation is further linked to respiratory health (Liddicoat et al. 2018), reduced asthma risk (Donovan et al. 2018), and greater diversity of skin and gut microbiota (Zhang et al. 2023), while residential green spaces have been associated with lower risk of atopic sensitization (Ruokolainen et al. 2015), reduced healthcare costs (Van Den Eeden et al. 2022), and even lower mortality (Rojas-Rueda et al. 2019, Bauwelinck et al. 2021).

In this pilot study, we investigated how urban green spaces and rewilding interventions influence microbial diversity on surrounding sealed surfaces. First, through a green space gradient experiment, we assessed whether microbial diversity associated with urban green spaces extends beyond green space boundaries onto adjacent impervious road surfaces. Samples were collected at four distances from the green space edges (0, 25, 50, and 100 m) to test how microbial richness and community composition change with increasing distance. Second, we conducted a rewilding experiment in the city center of Tampere, Finland, where a biodiverse urban garden was established within a paved and barren square in a former 19th-century industrial area. This intervention introduced diverse vegetation, compost-based growing media, and decaying wood with the aim of diversifying urban microbiota by enhancing local biodiversity. To investigate how the biodiversity garden affected the microbial communities of the surrounding urban space, environmental samples were collected before the rewilding and after the first growing season at three distances (0, 5, and 20 m). Samples were also collected from an adjacent control square which had existing vegetation. Together, these two approaches allowed us to evaluate both the influence of existing green spaces on nearby sealed surfaces and the potential of intentional rewilding to increase microbial diversity in highly impervious urban environments. We hypothesized that the existing urban green spaces and rewilding intervention increase microbial diversity and alter community structure on surrounding sealed surfaces. Specifically, we expected that (i) elevated microbial richness and diversity would be observed beyond the green spaces and that these measures would decline with distance, with distance being a stronger determinant of community composition than geographic location; and that (ii) rewilding a sealed urban square would increase microbial diversity, alter bacterial community composition, and increase the number of nodes and connections of bacterial co-occurrence networks compared to an unaltered control square.

## Materials and methods

### Green space gradient experiment

Five green spaces were selected for the green space gradient experiment. Four sites were located within urban areas in southern Finland: two in the Helsinki metropolitan area (population ∼1 583 000), one in Lahti (population ∼121 000), and one in Turku (population ∼206 000) (Supplementary Fig. 1). To provide a comparison beyond the urban context, one additional site was included at Arboretum Yltöinen, located ∼20 km from the city of Turku. The arboretum serves as a research area and gene bank for both domestic and foreign tree and shrub species. This site was chosen to examine whether microbial community patterns are specific to urban green spaces or whether the distance from green space remains the dominant explanatory factor, regardless of the surrounding landscape. Samples were collected in September 2021 from impervious surfaces (asphalt roads) at four distances from the green spaces: directly adjacent to the green space (0 m) and at 25, 50, and 100 m away.

### Rewilding experiment

**Figure 1:**
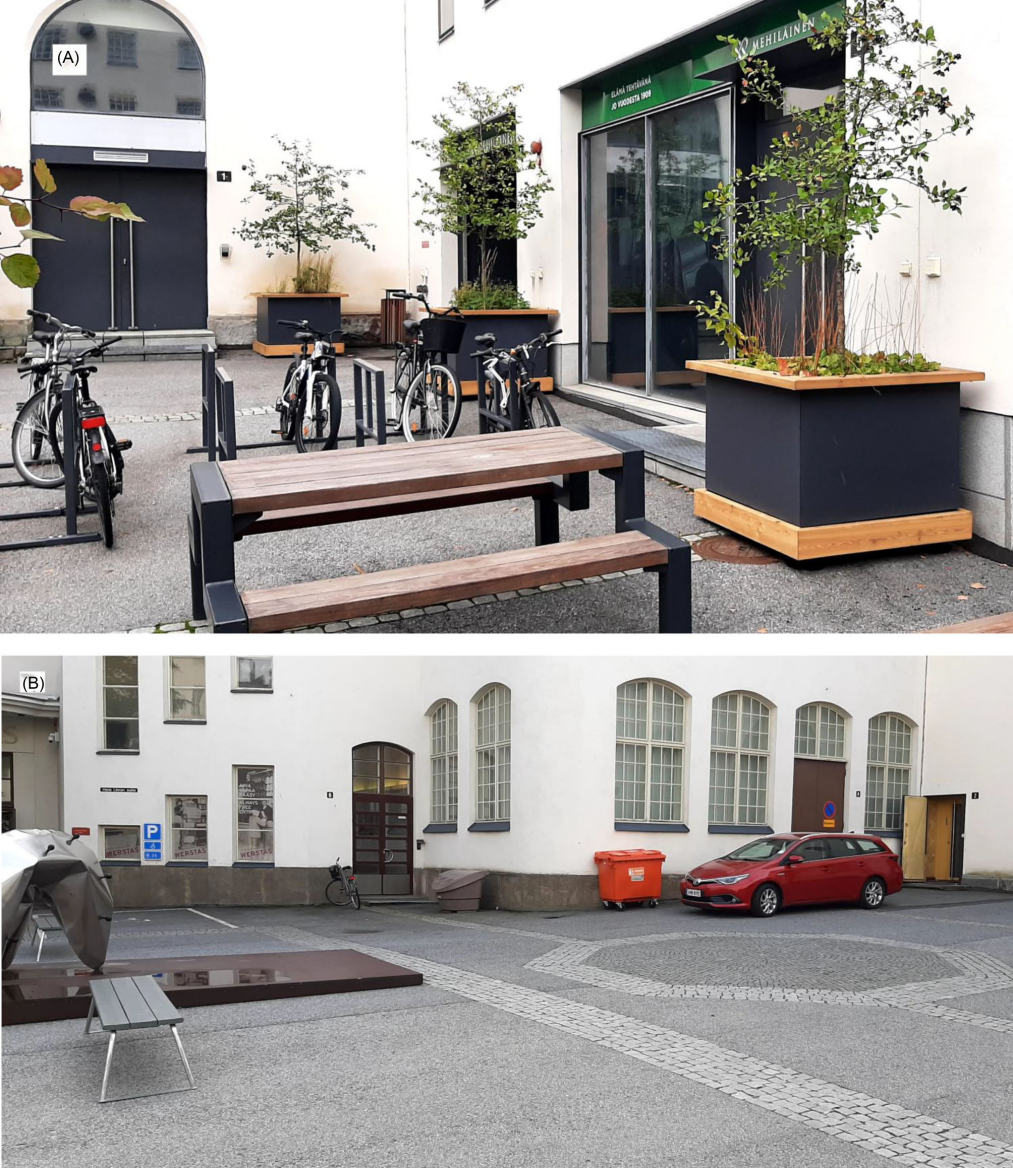
Pictures of the rewilding experiment’s control area. Control square in the fall of 2021 with (A) existing plant containers and (B) vegetation-free area.

**Figure 2:**
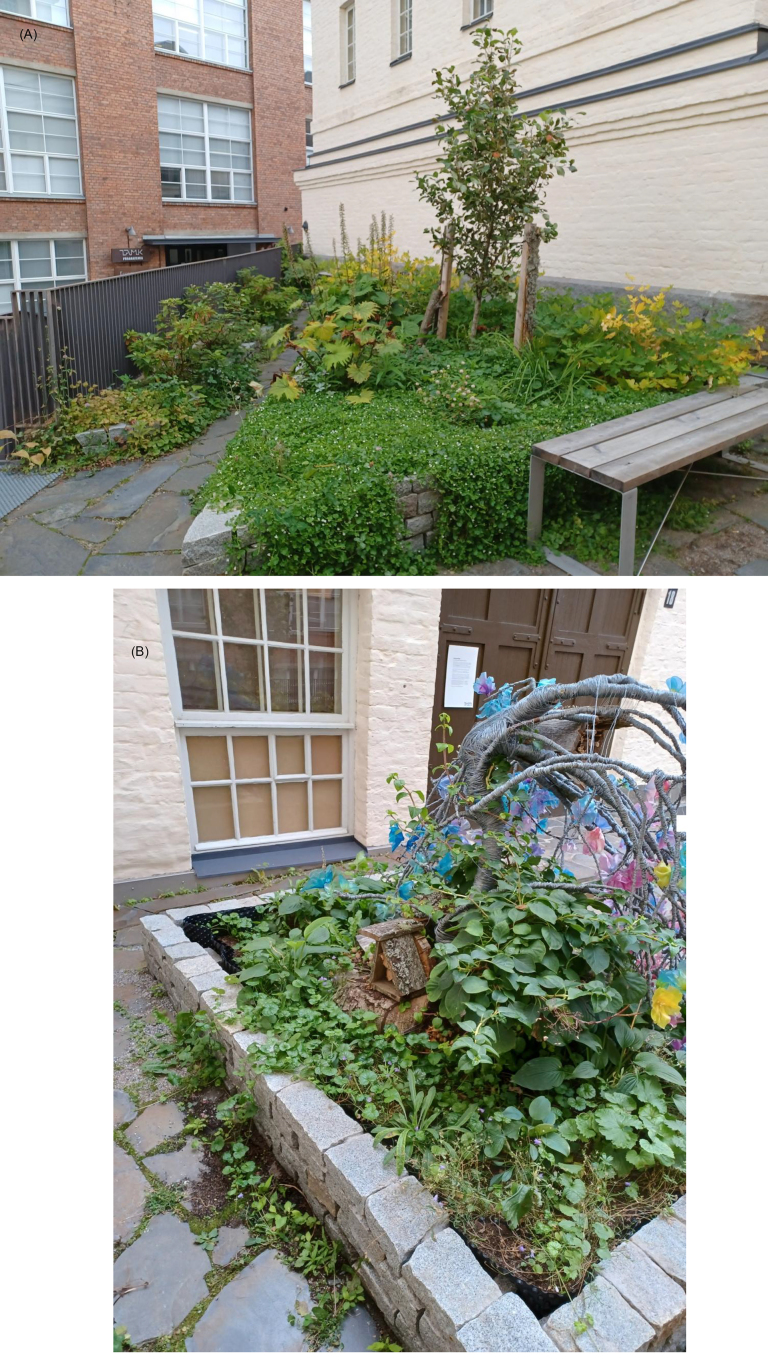
Pictures of the rewilding experiment’s intervention area. Intervention square in the fall of 2021 with (A) newly established garden beds containing annuals, perennials, and fruit trees (B) and decaying wood, and art.

The study area consists of two adjacent squares in the Finlayson district in the city of Tampere, Finland (population ∼250 000). The old factory area serves now as a daily workplace for office workers and offers entertainment, culture, and restaurants for residents and tourists. Both squares are completely enclosed with stone and brick walls but connected to each other and nearby squares and streets by passages. Motor vehicles enter both squares frequently. The control square (Väinö Linnan aukio) is entirely paved with asphalt and cobblestone and contains existing vegetation [one large European lime (*Tilia × europaea*), small greenhouse with perennial and annual plants, containers with small trees and annual plants; Fig. 1A and B]. The intervention square (Finlaysoninkuja) was entirely paved with asphalt and lacked any vegetation before the establishment of the biodiversity garden in the summer of 2021. The biodiversity garden is an ongoing and evolving project. The main goal is to accommodate a high level of biodiversity ranging from annual and perennial plants to microbes and animals, especially invertebrates. During the first summer in 2021, three garden beds and multiple containers were introduced into the area (Fig. 2A and B). Diverse soil microbial communities are supported by incorporating coarse woody debris (logs, branches, small standing snags), mulch, and compost into the garden beds. Environmental samples were taken from the control and intervention squares in the spring (end of May), before the establishment of the biodiversity garden, and fall (end of August) of 2021. In both squares, 15 samples were taken both in the spring and in the fall: five samples from the immediate vicinity of the containers or garden beds, five samples at 5 m from the containers or garden beds, and five samples at 20 m from the containers or garden beds (altogether 60 samples). The spring and fall samples were collected from the same spots and formed pairs.

### Spatial analysis

A multi-scale radius-based approach was utilized to examine the land use composition surrounding the study areas. By generating buffer zones with a 10–500 m radius, it captures the share of different land uses in the near proximity. The analysis calculates zonal statistics within each buffer using the 2018 Finnish CORINE Land Cover data and integrates high-resolution satellite imagery with various national datasets to produce both raster and vector databases at the resolution of 20 × 20 m. For each buffer zone, the method computes mean, sum, and count statistics of land use categories, providing a detailed characterization of the urban environment. In the green space gradient experiment, the main land cover category for the urban sampling sites was classified as discontinuous urban fabric and industrial or commercial units, covering 100% of the 10–500 m buffer zones. In the arboretum area, the dominant categories were mostly coniferous forest and nonirrigated arable land. In the rewilding experiment, impervious surfaces (e.g. buildings, roads) covered ~72% of the 200 m buffer zone, whereas vegetation cover was ~11%, surface water cover 16.5%, and open soil cover <0.5%.

### Sample collection and characterization

Sampling was executed on clear days without rain. Loose organic and inorganic material was collected into individual Ziplock bags with individually packed toothbrushes and disinfected disposable spoons. For each sample, the street surface was swept with a toothbrush for 30 s. Samples were stored at (−80°C until further processing.

In the rewilding experiment, moisture and dry matter contents of the environmental samples were determined by the thermogravimetric analysis (TGA). For this, samples from the same season (spring or fall) and distance (0, 5, or 20 m) were pooled as many of the individual samples did not meet the required sample mass of 5 g.

### Sample processing for microbial sequencing

Bacterial DNA was extracted from the environmental samples with the PowerSoil DNA Isolation Kit (Qiagen) as per the manufacturer’s standard protocol. DNA concentrations were quantified using the Quant-iT PicoGreen dsDNA Assay Kit (Thermo Fisher Scientific). The V4 region of the 16S rRNA gene was amplified with the 515F and 806R primers. Sterile water was used as a negative control during the DNA extraction and no-template control was used during the PCR. *Cupriavidus necator* JMP134 (DSM4058) was used as a positive control. All samples were sequenced at the Institute for Molecular Medicine Finland (FIMM, Helsinki, Finland) using the Illumina MiSeq platform (2 × 300 bp, V3 reagent kit).

In the rewilding experiment, real-time quantitative PCR (qPCR) of the 16S rRNA gene was performed using the LightCycler 96 Instrument (Roche). Primers pE (5´-AAA CTC AAA GGA ATT GAC GG-3′) and pF’ (5′-ACG AGC TGA CGA CAG CCA TG-3′) were utilized (Metabion) (Edwards et al. 1989). Each sample was amplified in triplicates in 20 μl reactions consisting of 10 μl of PowerTrack SYBR Green Master Mix (Thermo Fisher Scientific), 0.2 μl of BSA (20 mg/ml), 0.5 μl of each primer (10 μmol/l), 2.0 μl of the sample template, and 6.8 μl of water. Standard curves were included in all runs. The qPCR cycling consisted of the following steps: initial denaturation at 95°C for 2 min followed by 33 cycles of denaturation at 95°C for 10 s, annealing at 50°C for 20 s, and extension at 72°C for 30 s. Melting curve analysis of the amplicon was conducted with the following parameters: 95°C for 10 s, 65°C for 60 s, 97°C for 1 s, and 37°C for 30 s while continuously measuring the fluorescence signal. A mock community was used as a positive control (ZymoBIOMICS Microbial Community DNA Standard, 200 ng/20 µl, Zymo Research). The stock solution was diluted 1:100 in sterile water. Sterile water was used as a negative control.

### Bioinformatics

Raw sequence data were processed with the Divisive Amplicon Denoising Algorithm 2 (DADA2) pipeline (version 1.16) in the R environment (version 4.4.2) using the dada2 package (Callahan et al. 2016, R Core Team. 2020). Quality profiles were generated for raw sequences, and sequences were trimmed and filtered with the filterAndTrim function to remove low-quality bases, primers, and reads containing ambiguous nucleotides. Forward and reverse reads were merged using mergePairs. Identical sequences were dereplicated to reduce computational complexity while retaining abundance information for downstream denoising. Denoising and dereplication were carried out with default DADA2 parameters. Chimeras were detected and discarded with the removeBimeraDenovo function. Taxonomic assignments were performed against the SILVA reference database (version 138) using the assignTaxonomy function (Quast et al. 2013). Sequences classified as mitochondria or chloroplast were removed. Contaminant ASVs were removed as described by Roslund et al. (2021). Bacterial sequence data were deposited in IDA (rewilding experiment: https://doi.org/10.23729/614ab4e1-a354-4c83-b41c-13675a60a534, green space gradient experiment: https://doi.org/10.23729/fd-28d35ae8-a6de-39be-a9cf-5c495ba6c71c).

### Statistics

All statistical analyses and data visualizations were performed using the R statistical software environment (version 4.3.1, R Foundation, Vienna, Austria) (R Core Team. 2020). Following packages were used: vegan (version 2.5–7) (Oksanen et al. 2019) for diversity calculations and community composition analyses, phyloseq (version 1.38.0) (McMurdie and Holmes 2013) for community composition analyses, ggplot2 (version 3.3.5) (Wickham 2016) for community composition and diversity visualizations, and cooccur (version 1.3) (Griffith et al. 2016) and visNetwork (version 2.1.0) (Thieurmel 2021) for co-occurrence network analyses and visualizations.

To calculate the Shannon and Simpson diversity indices and observed richness, each sample was first subsampled to lowest sequence count to control for the varying number of sequences. The diversity indices were compared at different taxonomic levels (ASV, genus, family, order, class, phylum). Analyses were also conducted within the most abundant taxa of each taxonomic rank (relative abundance ≥1% across all samples). Diversity measures were compared using either *t*-tests (independent or paired), Mann–Whitney U test, Wilcoxon signed-rank test or linear mixed models. The *t*-tests were used when the data were normally distributed based on the Shapiro–Wilk test and the Mann–Whitney U test or Wilcoxon signed-rank test were used when the data were not normally distributed. *P*-values were corrected with the Benjamini–Hochberg correction to account for multiple testing (Benjamini and Hochberg 1995). Differences in the bacterial community compositions were analyzed at the ASV or genus level with the permutational analysis of variance (PERMANOVA), analysis of multivariate homogeneity of group dispersions (PERMDISP) followed by Tukey’s HSD test, and principal coordinates analysis. Center log ratio (CLR) transformed data and Aitchison distance were used for the calculations.

In the rewilding experiment, bacterial spring and fall co-occurrence networks were conducted at the genus level for both areas using subsampled binary presence-absence data, and basic topological parameters (nodes, edges, degree) were calculated from the networks. Co-occurrence networks can be a useful tool in identifying bacterial relationships (both negative and positive) and how these vary under different conditions, the role and importance of individual bacteria, and clusters of subcommunities.

## Results

### Green space gradient experiment

**Figure 3:**
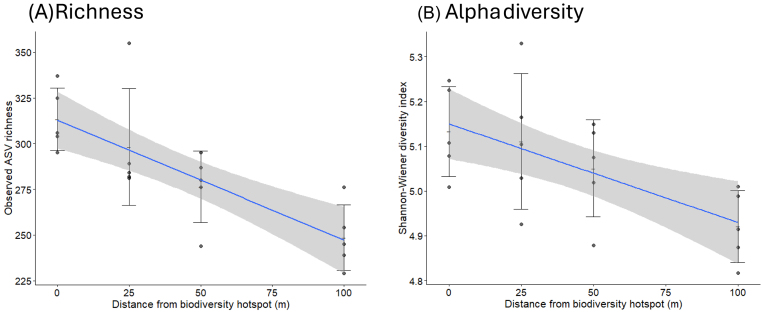
Bacterial richness and diversity along the green space distance gradient. (A) Bacterial richness (observed ASVs) was highest adjacent to the green space (0 m, *n* = 5) and declined with increasing distance (25–100 m, *n* = 5 per distance). (B) Alpha diversity (Shannon–Wiener diversity index) showed a similar distance-dependent decrease. Error bars indicate standard deviation of the mean.

Bacterial richness was highest directly adjacent to the green space (0 m) and declined progressively at 50 and 100 m (*P* = .05 and *P* = .0016, respectively; Fig. 3A). Alpha diversity showed a comparable distance-dependent pattern; however, a significant difference was observed only between 25 and 100 m (*P* > .0001; Fig. 3B).

**Figure 4:**
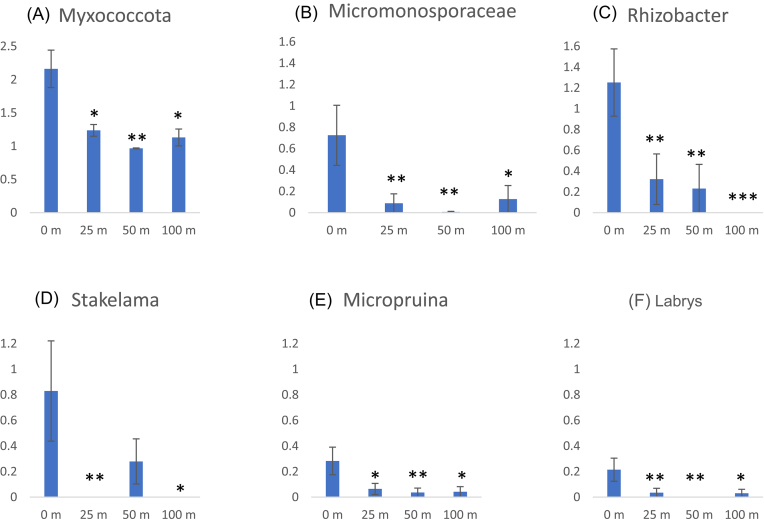
Relative abundance of bacterial taxa across the green space distance gradient. Bars show mean CLR-transformed abundances (± standard error) at 0, 25, 50, and 100 m from green spaces (*n* = 5 per distance). Taxa with significant differences across distances, including (A) phylum Myxococcota, (B) family Micromonosporaceae, and (C) genera *Rhizobacter*, (D) *Stakelama*, (E) *Micropruina*, and (F) *Labrys*, are presented. Significance levels indicate differences relative to the 0-m distance (*P < .05, **P < .01, ***P < .001).

At 0 m, the green spaces had a higher relative abundance of the phylum Myxococcota, family Micromonosporaceae, and genera *Rhizobacter, Stakelama, Micropruina*, and *Labrys* compared to other distances (Fig. 4; Supplementary Table 1). Relative abundance of *Rhizobacter*, in particular, decreased steadily across the distance gradient (Fig. 4C). The most common bacterial phyla for each distance were Proteobacteria, Actinobacteria, Bacteroidota, and Cyanobacteria (Supplementary Fig. 2).

Bacterial community composition at the ASV level at 50 m differed from that at 0 m, as indicated by PERMANOVA (*P* = .05; Supplementary Fig. 3A), and this effect was not attributable to differences in dispersion (PERMDISP: *P* > .5). Bacterial communities at 50 and 100 m formed a tightly clustered group, despite originating from different locations (Supplementary Fig. 3A). Bacterial community dispersion was greater at 25 m compared to 100 m (PERMDISP: *P* = .001). Community composition in the arboretum research area differed from the green spaces located in cities (*P* = .03; Supplementary Fig. 3B).

### Rewilding experiment

**Figure 5:**
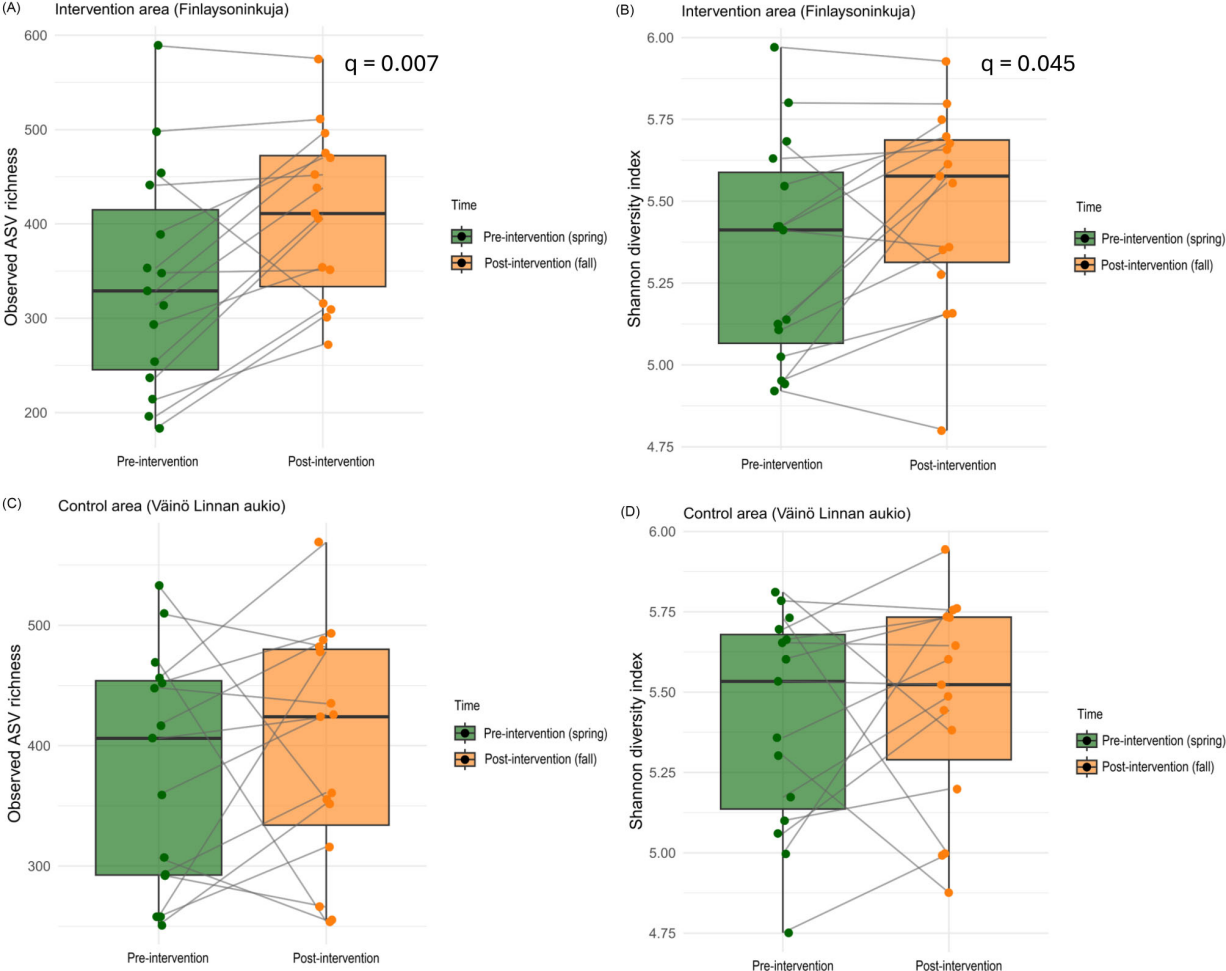
Bacterial richness and diversity at the ASV level in the intervention and control areas of the rewilding experiment. In the intervention area, the (A) observed richness (*n* = 30) and (B) Shannon diversity (*n* = 30) of the environmental bacteria were higher in the fall than in the spring. In the control area, no differences in the (C) observed richness (*n* = 30) or (D) Shannon diversity (*n* = 30) were detected. Connected dot plot show medians (thick line), upper and lower hinges (box), values 1.5 times the interquartile range (whiskers), points represent individual samples, and lines connect paired spring and fall observations within each site. *Q*-values represent *P*-values after correction for multiple testing using the Benjamini–Hochberg correction.

Real-time quantitative PCR revealed that the amount of 16S rRNA gene copies increased after the garden had been established only in the intervention area’s 0 m samples (*P* < .023; Supplementary Table 2). The observed richness and alpha diversity of the urban bacterial community in the intervention area increased after the establishment of the garden (ASV level: *P* = .006 and *P* = .045, respectively; Fig. 5A and B). The observed richness and Shannon diversity in the intervention area were higher in the fall also at the genus, family, order, class, and phylum levels (Table 1A and B). The Simpson diversity followed a similar pattern, except for the phylum, order, and ASV levels (Table 1C). The richness and Shannon diversity of many of the major phyla and of the classes Bacilli and Gammaproteobacteria increased from spring to fall in the intervention area (Table 1). The richness and diversity changes were observed when all samples from different distances were pooled. No differences were observed when the distances 0–20 m were analyzed separately. In the control area, no differences in the environmental bacterial diversities between the seasons were observed (Fig. 5C and D; Supplementary Table 3). No significant changes were observed for organic matter or moisture content (Supplementary Table 4).

**Table 1:** Bacterial richness and alpha diversity in the intervention area of the rewilding experiment (*n* = 30). (A) Observed richness and (B) Shannon and (C) Simpson diversities of the environmental bacteria in the intervention area were compared between the spring and fall seasons using paired tests. Observed richness (mean ± standard deviation), Shannon diversity (mean ± standard deviation), Simpson diversity (mean ± standard deviation), *P*-values, and *Q*-values are given for taxonomic groups. *Q*-values represent *P*-values after correction for multiple testing using the Benjamini–Hochberg correction. Bold values indicate statistically significant results (p < 0.05).

	Measure	Baseline, spring		Fall			
(A) Observed richness		Mean	SD	Mean	SD	*P*	*Q*
	ASV	339.47	118.04	409.13	89.35	**.006**	**.007**
	Genus	130.73	32.23	149.53	34.63	**.047**	**.047**
	Family	89.47	20.68	114.47	23.28	**<.0001**	**<.001**
	Order	63.93	15.22	89.27	19.20	**<.0001**	**<.001**
	Class	33.73	8.20	43.67	10.52	**<.0001**	**<.001**
	Phylum	17.13	2.77	21.87	4.60	**.001**	**.002**
	Phylum_Verrucomicrobiota	8.67	5.77	17.60	7.75	**.005**	**.018**
	Phylum_Proteobacteria	112.73	33.58	119.73	32.36	.423	.508
	Phylum_Planctomycetota	18.93	12.94	29.80	11.91	**.004**	**.020**
	Phylum_Myxococcota	9.67	6.90	14.47	5.71	**.012**	**.028**
	Phylum_Gemmatimonadota	4.73	2.84	7.13	4.41	**.022**	**.034**
	Phylum_Firmicutes	2.27	2.71	22.53	26.45	**.003**	**.015**
	Phylum_Deinococcota	6.53	3.11	3.53	2.07	**.008**	**.018**
	Phylum_Cyanobacteria	9.00	7.71	3.73	2.60	**.002**	**.015**
	Phylum_Chloroflexi	14.60	12.56	24.13	15.00	**.033**	**.049**
	Phylum_Bacteroidota	60.47	25.66	59.33	16.78	.898	.898
	Phylum_Actinobacteriota	53.33	21.07	51.53	18.80	.739	.806
	Phylum_Acidobacteriota	17.13	14.04	21.33	11.39	.209	.278
	Class_Gammaproteobacteria	42.33	13.98	52.53	12.56	**.016**	**.043**
	Class_Bacilli	1.87	2.39	11.40	12.69	**.004**	**.030**
	Class_Alphaproteobacteria	70.13	22.06	66.93	23.72	.635	.781
(B) Shannon diversity index	ASV	5.34	0.34	5.49	0.30	**.045**	**.045**
	Genus	4.14	0.40	4.38	0.26	**.031**	**.038**
	Family	3.60	0.41	3.91	0.29	**.001**	**.006**
	Order	3.12	0.38	3.41	0.28	**.003**	**.010**
	Class	2.19	0.38	2.42	0.30	**.011**	**.022**
	Phylum	1.68	0.34	1.87	0.20	**.016**	**.024**
	Phylum_Verrucomicrobiota	1.68	0.69	2.45	0.48	**.001**	**.004**
	Phylum_Proteobacteria	4.28	0.32	4.33	0.30	.553	.663
	Phylum_Planctomycetota	2.44	1.00	3.03	0.36	**.005**	**.016**
	Phylum_Myxococcota	1.78	0.90	2.29	0.43	**.041**	.071
	Phylum_Gemmatimonadota	1.19	0.64	1.51	0.73	.073	.109
	Phylum_Firmicutes	0.51	0.60	1.93	1.32	**.003**	**.013**
	Phylum_Deinococcota	1.51	0.43	0.91	0.60	**.006**	**.018**
	Phylum_Cyanobacteria	1.68	0.65	0.82	0.66	**.001**	**.004**
	Phylum_Chloroflexi	2.12	0.87	2.60	0.75	**.014**	**.028**
	Phylum_Bacteroidota	3.69	0.44	3.71	0.30	.888	.888
	Phylum_Actinobacteriota	3.55	0.37	3.50	0.39	.607	.663
	Phylum_Acidobacteriota	2.31	0.69	2.57	0.60	.188	.251
	Class_Gammaproteobacteria	3.28	0.37	3.51	0.27	**.021**	.055
	Class_Bacilli	0.40	0.53	1.47	1.11	**.005**	**.027**
	Class_Alphaproteobacteria	3.85	0.31	3.78	0.36	.482	.593
(C) Simpson diversity index	ASV	0.99	0.00	0.99	0.00	.359	.359
	Genus	0.97	0.02	0.98	0.01	**.041**	.076
	Family	0.95	0.02	0.97	0.01	**.015**	.076
	Order	0.92	0.03	0.94	0.02	.064	.076
	Class	0.83	0.06	0.86	0.04	**.034**	.054
	Phylum	0.71	0.11	0.76	0.05	.055	.076
	Phylum_Verrucomicrobiota	0.73	0.20	0.87	0.07	**.007**	.075
	Phylum_Proteobacteria	0.98	0.01	0.98	0.01	.679	.720
	Phylum_Planctomycetota	0.83	0.26	0.94	0.02	**.012**	.075
	Phylum_Myxococcota	0.85	0.12	0.86	0.06	.720	.720
	Phylum_Gemmatimonadota	0.66	0.24	0.67	0.28	.599	.720
	Phylum_Firmicutes	0.55	0.37	0.73	0.30	.055	.133
	Phylum_Deinococcota	0.72	0.11	0.49	0.29	**.022**	.086
	Phylum_Cyanobacteria	0.73	0.16	0.56	0.33	.121	.241
	Phylum_Chloroflexi	0.80	0.24	0.87	0.12	.055	.133
	Phylum_Bacteroidota	0.96	0.02	0.97	0.01	.679	.720
	Phylum_Actinobacteriota	0.96	0.01	0.96	0.02	.443	.665
	Phylum_Acidobacteriota	0.86	0.08	0.88	0.10	.524	.720
	Class_Gammaproteobacteria	0.95	0.02	0.96	0.02	.083	.333
	Class_Bacilli	0.55	0.39	0.72	0.29	.249	.442
	Class_Alphaproteobacteria	0.97	0.01	0.97	0.01	.398	.638

The most common bacterial phyla in both areas were Proteobacteria, Actinobacteria, Bacteroidetes, Cyanobacteria, and Acidobacteria (Supplementary Fig. 4). According to PERMANOVA, the post-rewilding fall bacterial community at the genus level in the intervention area differed from the pre-rewilding spring community and from the control area’s communities (Table 2; Supplementary Fig. 5). The spring bacterial community in the control area did not differ from the fall community or from the intervention area’s spring community (Table 2). These differences were not attributable to differences in dispersion (PERMDISP: *P* > .5).

**Table 2:** PERMANOVA results for the rewilding experiment.

Group 1 (*n* = 15)	Group 2 (*n* = 15)	*F*	*R* ^2^	*P*	q
Intervention fall	Intervention spring	4.774	0.146	**.004**	**.012**
Intervention fall	Control fall	2.976	0.096	**.011**	**.017**
Intervention fall	Control spring	5.096	0.154	**.001**	**.006**
Intervention spring	Control fall	3.037	0.098	**.009**	**.017**
Intervention spring	Control spring	1.304	0.0.45	.207	.207
Control fall	Control spring	1.802	0.060	.055	.066

*F*-values, *R*^2^-values, *P*-values, and *Q*-values are given. *Q*-values represent *P*-values after correction for multiple testing using the Benjamini–Hochberg correction. Bold values indicate statistically significant results (p or q < 0.05).

**Figure 6:**
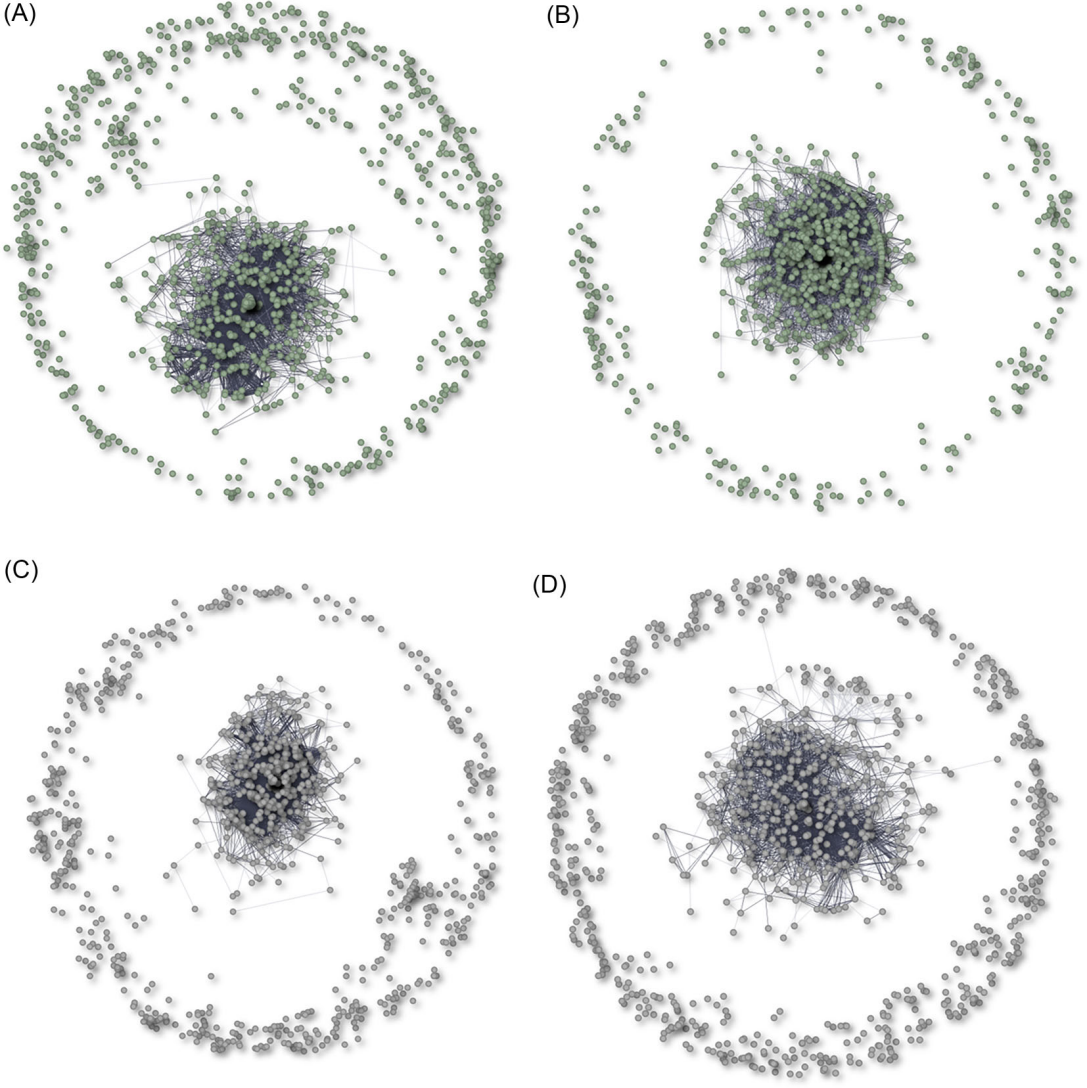
Bacterial co-occurrence networks of the intervention and control areas in the rewilding experiment. Co-occurrence network plots of the environmental bacteria at the genus level in the intervention area in the (A) spring (*n* = 15) and (B) fall (*n* = 15) and in the control area in the (C) spring (*n* = 15) and (D) fall (*n* = 15) are shown.

Spring and fall bacterial co-occurrence networks were constructed for the intervention and control areas at the genus level (Fig. 6). The number of nodes and edges (connections) and the average degree (edges per node) of the intervention area’s bacterial co-occurrence network was higher in the fall, after the establishment of the garden, than in the spring (Table 3). In the control area network, the number of nodes and edges and the average degree decreased from spring to fall (Table 3). In both areas, the proportion of positive correlations decreased whereas the proportion of negative correlations increased. After the establishment of the garden, the intervention area bacterial co-occurrence network had more nodes and edges than the control area network.

**Table 3:** Basic topological parameters of the bacterial co-occurrence networks at the genus level in the intervention and control areas of the rewilding experiment.

Location	Season	Nodes	Edges	Average degree	Positive correlations	Positive correlations %	Negative correlations	Negative correlations %
Interventionarea	Spring (*n* = 15)	400	4065	20.3	3810	93.73%	255	6.27%
	Fall (*n* = 15)	635	15 843	49.4	12 715	80.26%	3128	19.74%
Controlarea	Spring (*n* = 15)	412	10 023	48.6	9420	93.98%	603	6.02%
	Fall (*n* = 15)	401	3645	18.2	2958	81.15%	687	18.85%

## Discussion

Our pilot study provides evidence that both existing green spaces and rewilding interventions shape microbial communities on sealed urban surfaces. In the green space gradient experiment, bacterial richness and diversity were the highest directly adjacent to the green spaces and declined with increasing distance. The bacterial communities at 50 and 100 m formed a tightly clustered group despite originating from different cities, suggesting that distance to the green space was a stronger determinant of microbial community composition than geographic location. In the rewilding experiment, the establishment of a biodiversity garden in a former industrial area increased bacterial alpha diversity, altered community composition, and increased the number of co-occurrence network nodes and edges in the intervention square, while no comparable changes were observed in the control square. Together, these findings support our overarching hypothesis that green spaces and rewilding both enrich microbial diversity and restructure microbial communities in urban environments.

Based on the green space gradient experiment, the effects’ spatial extent appears to be small, with benefits concentrated close to the green spaces. Bacterial richness and diversity on impervious surfaces dropped quickly after the green space edge, in general after 50 m and with some putatively beneficial taxa already at 25 m. At the same time, we observed little change in overall bacterial community composition across distances, implying that microbiomes on hard, impermeable substrates are relatively homogeneous at the broad compositional level, likely shaped by shared microclimatic and physicochemical constraints of those surfaces. This inference is tentative: the study included only five green spaces, so expanding the number and types of sites, adding multi-directional transects (to account for wind and street-canyon effects), and controlling for covariates such as traffic load, surface age, and maintenance will improve power and interpretability. Despite broad compositional similarity across distances, several taxa linked to possible ecosystem services showed clear distance‐related shifts in their relative abundance, being most abundant adjacent to the green spaces and reduced already at 25 m from the edge. Myxococcota are known for their predatory lifestyle and ability to produce a great variety of secondary metabolites making them potential biological control agents capable of regulating soil microbial communities (Contreras-Moreno et al. 2024, Wang et al. 2024). Micromonosporaceae are renowned for their production of bioactive metabolites and role in biocontrol, plant-growth promotion, root ecology, and breakdown of cellulosic materials (Hirsch and Valdés 2010, Hifnawy et al. 2020, Nouioui et al. 2025). While *Rhizobacter* are commonly recognized as plant pathogens, some species are capable of degrading plastics and rubber (Goto and Kuwata 1988, Imai et al. 2013, Sagong et al. 2021). *Micropruina* species isolated from sludge have been observed to possess enhanced biological phosphorus removal activity (Shintani et al. 2000). Lastly, *Labrys* species have been observed to degrade a wide array of pollutants and pharmaceuticals (Aguilar-Romero et al. 2025). While ecosystem services were not measured in this study, the findings suggest the hypothetical “microbial service footprint” of urban green spaces is very local (∼25–50 m). A practical implication is that to maximize microbial diversity and possible accompanying microbial-mediated ecosystem services, such as plant support, pollutant attenuation, and human exposure benefits, green infrastructure should be proximate and frequent: vegetated verges, pocket gardens, hedgerows near footpaths, and plantings near buildings.

In the rewilding experiment, both the spring and fall sampling in the control area resulted in mostly dust, gravel, and sand being collected, even though the area had existing vegetation, indicating comprehensively sealed soil and no spillover from planters. The spring samples from the intervention area had a similar inorganic makeup, especially in the immediate vicinity of the future garden beds where the area was completely barren. The organic content and number of 16S rRNA gene copies in the intervention area’s 0 m spring samples were the lowest of all samples (Supplementary Tables 2 and 4). Samples taken after the establishment of the garden seemed to contain visibly more organic material, which was confirmed by the TGA (Supplementary Table 4). These differences were echoed in the diversity analyses: bacterial alpha diversities and 16S rRNA gene copies increased only in the intervention area and the composition of the intervention area’s bacterial community was altered after the rewilding. No changes were observed in the control area with existing vegetation, suggesting that the increasing diversities were not a result of the season. These findings are noteworthy, since soil microbial communities in urban green spaces have been observed to become more homogenous on a global scale (Delgado-Baquerizo et al. 2021). The green space gradient experiment results support this pattern of homogenization, as the community compositions of samples from different urban locations did not differ from each other. The rewilding results suggest that rewilding can counteract this trend as the intervention led to increased differentiation and diversity of the bacterial communities within the urban space rather than homogenization. It is worth noting that the streets of the intervention area are cleaned and painted every summer as a community art project (Supplementary Fig. 6). As the painting and cleaning of the previous year’s street art took place after the garden had been established, it is unlikely that the intervention area’s fall samples consisted merely of growing media that had spilled onto the streets during the spring planting. Instead, the spreading of the organic material and microbes from the garden beds seems to have occurred throughout the growing season.

The alpha diversity analyses revealed that the biodiversity garden increased the intervention area’s bacterial diversity at all taxonomic levels. Some studies have found that bacterial richness can be high under anthropogenic stressors (e.g. pollution, drought, heat) which are common in urban areas (Reese et al. 2016, Christel et al. 2023), but the results of this experiment demonstrate how deliberate increase in urban microbial diversity can be achieved using nature-based solutions and materials. Increased bacterial richness in urban areas might be important in the context of human commensal microbiota and immune regulation as living environments with high bacterial richness and Shannon diversity have been associated with lower risk of some immune-mediated noncommunicable diseases, such as allergic rhinitis and atopy (Ege et al. 2011, 2012, Valkonen et al. 2015, Hyytiäinen et al. 2021). In addition to the overall diversity, the diversity of multiple phyla and classes, even health-related, increased. For example, Gammaproteobacteria have been associated with elevated levels of immunomodulatory molecules in the blood and lower risk of atopic sensitization and allergic conditions (Hanski et al. 2012, Fyhrquist et al. 2014, Roslund et al. 2020, 2022). More studies involving human participants are needed to test the assumption that rewilding interventions can also influence human commensal microbial communities and, in turn, provide measurable benefits for immune regulation.

In the rewilding experiment, the possible interactions between the bacteria changed, too. In the control area, the number of nodes (individual taxa) and edges (correlations, interconnections) of the genus-level co-occurrence network decreased, whereas in the intervention area the network contained more nodes and edges after the garden was established. This is in accordance with the increased genus-level richness and Shannon diversity in the intervention area; it is logical that when the number of bacterial taxa increases, so do the interactions between them. It has been suggested that the nature of the interconnections matters more than the mere number of them. In both areas, the relative abundance of positive correlations dropped, which may reflect reduced environmental stress (Hoek et al. 2016, Velez et al. 2018, Piccardi et al. 2019). A positive correlation can mean that organisms truly cooperate or that they simply favor similar environmental conditions, and, in a similar manner, a negative correlation can imply that the organisms thrive in different environments or compete (Das et al. 2018). Stress can also dismember microbial networks, and drought, for example, has been found to decrease the total number of nodes and edges in bacterial networks (Vries de et al. 2018, Gao et al. 2022). Interestingly, positive interconnections have been observed to dominate among bacteria that inhabit plant roots, and that this cooperation increases both bacterial abundance and plant health (Ren et al. 2015, Durán et al. 2018). This suggests that rewilding may restructure microbial networks toward greater complexity, but not necessarily toward cooperative interactions.

Our study has several limitations. First, the rewilding experiment included only one intervention site and the green space gradient experiment was limited to five sites, all located in Finland. While this focused design allowed for controlled comparisons, it limits the generalizability of our findings to other geographic regions and climatic contexts. Microbiota responses to rewilding may differ substantially between boreal systems such as those studied here and temperate, Mediterranean, or tropical environments, where baseline biodiversity, species interactions, soil composition, and disturbance regimes vary. Similarly, climatic factors such as temperature, precipitation patterns, seasonality, and length of the growing season can influence ecosystem processes and the outcomes of urban rewilding. Urban form and land-use history may also differ across regions, further shaping microbiota trajectories. Additionally, as both the spatial and temporal proximity of the samples was high, the risk of pseudoreplication is also considerable. Future studies should therefore include a larger number of sampling sites across diverse geographic regions and climatic zones and extend sampling over multiple years to better capture temporal variability and improve the broader applicability of the results. Second, the use of low-biomass samples, in our case inorganic gravel and sand, poses challenges related to amplification bias and contamination. Despite these constraints, our results indicate that green spaces allow the dispersion of microbiota onto adjacent impermeable surfaces, but only over a very short range. Rewilding urban environments with multiple small biodiverse patches may be a practical way to increase local microbiota diversity. One limitation is also that our study focused only on microbial communities on sealed surfaces. We observed increased microbial diversity at street level, and through, for example, shoes, humans might take some of these diverse microbial communities home, but the occurrence of this is unknown. Microbial exposure also occurs via air, and vegetation shapes airborne microbial communities and this way even the human microbiota (Mhuireach et al. 2016, Selway et al. 2020). Future work should include ambient-air sampling and parallel skin and airway sampling to assess the effects of rewilding on outdoor and indoor aerobiomes and human microbiota. Together with source-tracking, these would allow us to test causality and to quantify how design choices (canopy complexity, plant diversity) increase, or fail to increase, microbial life within the urban fabric.

While the rewilding experiment focused on the biodiversity garden’s effects in diversifying urban microbiota, the garden was designed and established for all kinds of life forms. Already after the first growing season, a high variety of animals, both invertebrates and vertebrates, had found their way in. More than 20 different pollinator species were observed during the fall sampling, and after the first winter, many of the holes made on the dead trees were occupied by solitary bees and wasps. The same dead trees also hosted different wood-decay fungi. As the garden is being improved, it is important to monitor how its biodiversity evolves at different levels and how these changes affect the microbial communities. Biodiverse urban green spaces should be preserved, enhanced, and expanded to support planetary health on multiple levels.

## Conclusion

This pilot study shows that rewilding city squares increases microbial richness and diversity on sealed surfaces, but the effect is highly localized. The microbial communities directly adjacent to existing green spaces include higher relative abundance of bacteria linked to possible ecosystem services (e.g. biocontrol, pollutant degradation, nutrient cycling), indicating that green spaces have the potential to strengthen local environmental functions where people live and work. Urban design should therefore favor frequent, proximate plantings, such as pocket gardens with compost-enriched growing medium, diverse native plantings and decaying deadwood, vegetated verges, and hedgerows. The environmental gains of rewilding may yield secondary human health benefits via greater exposure to diverse environmental microbes, but this remains to be confirmed. Future studies should pair surface sampling with aerobiome sampling alongside targeted skin and airway measures to determine whether rewilding enhances beneficial microbial exposure for urban dwellers.

## Supplementary Material

qvag020_Supplemental_File

## Data Availability

Bacterial sequence data for rewilding experiment is available at https://doi.org/10.23729/614ab4e1-a354-4c83-b41c-13675a60a534 and for green space gradient experiment at https://doi.org/10.23729/fd-28d35ae8-a6de-39be-a9cf-5c495ba6c71c.
