# Supplementary material for: Sustainable rewilding of urban spaces increases microbial diversity
Source: Sustain Microbiol. 2026 May 22;3(2):qvag020. doi: 10.1093/sumbio/qvag020 (PMC13375368; doi:10.1093/sumbio/qvag020)

Supplmementary Tables and Figures

Supplement to: Mika Saarenpää, Juulia Manninen, Damiano Cerrone, Juho Rajaniemi, Marja I. Roslund*, Aki Sinkkonen*. **Sustainable Rewilding of Urban Spaces Increases Microbial Diversity.**

Supplementary Table 1. Linear mixed model (LMM) results for differences between distances 0, 25, 50 and 100 m (n = 5 per distance; total n = 20). Data is presented as mean ± standard deviation (sd) for CLR-transformed abundances. Probability *p*-values and fixed R squared (R2) values are given for the models.

	Distance 0 m		Distance 25 m		Distance 50 m		Distance 100 m		*p*			
	Mean	sd	Mean	sd	Mean	sd	Mean	sd	**0 vs. 25 m**	**0 vs. 50 m**	**0 vs. 100 m**	Fixed R2
Phyla Myxococcota	2.16	0.94	1.24	0.71	0.97	0.73	1.13	0.49	**0.035**	**0.009**	**0.035**	0.33
Family Micromonos-poraceae	0.72	0.69	0.09	0.22	0.01	0.01	0.13	0.25	**0.006**	**0.003**	**0.022**	0.40
Genus Rhizobacter	1.25	0.79	0.32	0.60	0.23	0.52	0.00	0.00	**0.004**	**0.002**	**<0.001**	0.45
Genus Stakelama	0.83	0.96	0.00	0.00	0.28	0.39	0.00	0.00	**0.010**	**0.101**	**0.021**	0.33
Genus Micropruina	0.28	0.26	0.06	0.11	0.04	0.08	0.04	0.08	**0.016**	**0.008**	**0.017**	0.35
Genus Labrys	0.21	0.22	0.03	0.08	0.00	0.00	0.03	0.06	**0.008**	**0.005**	**0.038**	0.35

Supplementary Table 2. Differences in 16S rRNA gene copy numbers between spring and fall were tested within each distance group using paired t-tests. Tests were conducted separately for intervention and control areas (n = 5 paired samples per distance within each area; total per area: n = 15 in spring and n = 15 in fall). Copy numbers are presented for individual samples, and p-values correspond to comparisons within distance groups.

Location	Distance (m)	Sample	Season	16S rRNA gene copies/µl	*p*
Intervention area	0	1	Spring	16	0.0228
		1	Fall	6674000	
		2	Spring	21	
		2	Fall	214100	
		3	Spring	12	
		3	Fall	5887000	
		4	Spring	42280	
		4	Fall	5398000	
		5	Spring	3982000	
		5	Fall	6987000	
	5	1	Spring	8996000	0.0881
		1	Fall	756800	
		2	Spring	29980000	
		2	Fall	28600000	
		3	Spring	10690000	
		3	Fall	6941000	
		4	Spring	9732000	
		4	Fall	2859000	
		5	Spring	7430000	
		5	Fall	8474000	
	20	1	Spring	8318000	0.2205
		1	Fall	77040000	
		2	Spring	1197000	
		2	Fall	5189000	
		3	Spring	9544000	
		3	Fall	88420000	
		4	Spring	50390000	
		4	Fall	8142000	
		5	Spring	5717000	
		5	Fall	61940000	
Control area	0	1	Spring	97340000	0.9764
		1	Fall	42210000	
		2	Spring	7185000	
		2	Fall	4844000	
		3	Spring	63420000	
		3	Fall	89750000	
		4	Spring	32410000	
		4	Fall	24300000	
		5	Spring	71630000	
		5	Fall	113500000	
	5	1	Spring	72680000	0.6972
		1	Fall	5872000	
		2	Spring	7254000	
		2	Fall	79320000	
		3	Spring	5196000	
		3	Fall	3998000	
		4	Spring	1959000	
		4	Fall	47060000	
		5	Spring	370300	
		5	Fall	705000	
	20	1	Spring	5543000	0.8646
		1	Fall	46850000	
		2	Spring	38780000	
		2	Fall	56670000	
		3	Spring	2784000	
		3	Fall	52360000	
		4	Spring	49810000	
		4	Fall	879700	
		5	Spring	43010000	
		5	Fall	1940000	

Supplementary Table 3. A) Observed richness and B) Shannon and C) Simpson diversities in the control area were compared between the spring (n = 15) and fall seasons (n = 15) using paired tests. Observed richness (mean ± standard deviation), Shannon diversity (mean ± standard deviation), Simpson diversity (mean ± standard deviation), and *p*-values are given for taxonomic groups. Q-values are not given as no *p*-value remained significant after correction.

	Measure	Baseline, spring		Fall		Wilcox statistics	
		Mean	sd	Mean	sd	statistic	*p*
A) Observed richness	ASV_Observed	380.60	97.59	396.93	97.58	79	0.303
	Genus_Observed	146.20	33.23	140.47	25.61	44	0.378
	Family_Observed	94.20	20.81	96.87	18.78	68	0.670
	Order_Observed	66.53	11.58	69.13	12.20	75.5	0.394
	Class_Observed	33.47	5.66	35.67	6.54	72.5	0.220
	Phylum_Observed	17.27	1.58	17.67	2.26	58	0.751
	Phylum_Verrucomicrobiota_observed	11.40	5.28	13.27	8.04	71.5	0.245
	Phylum_Proteobacteria_observed	106.47	31.13	107.53	31.27	63.5	0.865
	Phylum_Planctomycetota_observed	22.13	6.93	30.60	16.13	89	0.105
	Phylum_Myxococcota_observed	12.67	10.91	15.33	10.09	82	0.222
	Phylum_Gemmatimonadota_observed	7.53	3.91	9.73	6.53	81	0.243
	Phylum_Firmicutes_observed	1.87	2.13	1.60	1.99	28.5	0.720
	Phylum_Deinococcota_observed	5.73	2.79	6.00	3.85	66.5	0.733
	Phylum_Cyanobacteria_observed	17.40	9.98	22.53	13.91	86	0.147
	Phylum_Chloroflexi_observed	20.40	12.22	18.60	9.74	41.5	0.510
	Phylum_Bacteroidota_observed	68.00	13.19	60.67	12.29	40.5	0.280
	Phylum_Actinobacteriota_observed	59.53	25.89	60.93	26.53	54	0.950
	Phylum_Acidobacteriota_observed	20.13	8.59	19.33	7.88	55.5	0.820
	Class_Gammaproteobacteria_observed	38.20	14.16	40.33	14.06	64.5	0.820
	Class_Bacilli_observed	1.33	1.88	1.53	2.00	23.5	0.952
	Class_Alphaproteobacteria_observed	67.93	17.82	66.60	25.46	56	0.842
B) Shannon diversity Index	ASV_Shannon	5.41	0.33	5.47	0.32	75	0.421
	Genus_Shannon	4.27	0.34	4.31	0.23	66	0.762
	Family_Shannon	3.72	0.31	3.79	0.23	76	0.389
	Order_Shannon	3.22	0.21	3.30	0.18	80	0.277
	Class_Planctomycetes_shannon	2.36	0.54	2.64	0.54	82	0.229
	Phylum_Planctomycetota_shannon	2.73	0.44	3.01	0.55	87	0.135
	Phylum_Verrucomicrobiota_shannon	1.95	0.59	2.13	0.61	88	0.121
	Phylum_Shannon	1.88	0.12	1.95	0.13	95	**0.048**
	Phylum_Proteobacteria_shannon	4.22	0.32	4.23	0.32	72	0.524
	Phylum_Myxococcota_shannon	1.85	0.98	2.18	0.74	87	0.135
	Phylum_Gemmatimonadota_shannon	1.62	0.63	1.74	0.77	68	0.679
	Phylum_Firmicutes_shannon	0.46	0.65	0.37	0.59	18	0.636
	Phylum_Deinococcota_shannon	1.36	0.46	1.39	0.71	65	0.804
	Phylum_Cyanobacteria_shannon	2.19	0.76	2.46	0.85	77	0.359
	Phylum_Chloroflexi_shannon	2.33	1.02	2.37	0.83	53	0.720
	Phylum_Bacteroidota_shannon	3.85	0.19	3.74	0.21	35	0.169
	Phylum_Actinobacteriota_shannon	3.63	0.51	3.65	0.54	61	0.978
	Phylum_Acidobacteriota_shannon	2.52	0.47	2.50	0.43	62	0.934
	Class_Gammaproteobacteria_shannon	3.12	0.39	3.22	0.36	71	0.561
	Class_Bacilli_shannon	0.32	0.57	0.32	0.59	18	1.000
	Class_Alphaproteobacteria_shannon	3.84	0.30	3.78	0.40	49	0.561
C) Simpson diversity Index	ASV_Simpson	0.99	0.01	0.99	0.00	72	0.524
	Genus_Simpson	0.97	0.02	0.98	0.01	72	0.524
	Family_Simpson	0.96	0.01	0.96	0.01	67	0.720
	Order_Simpson	0.94	0.01	0.94	0.01	71	0.561

	Class_Planctomycetes_simpson	0.85	0.11	0.90	0.05	78	0.330
	Phylum_Planctomycetota_simpson	0.91	0.06	0.93	0.04	83	0.208
	Phylum_Verrucomicrobiota_simpson	0.78	0.14	0.83	0.10	95	**0.048**
	Phylum_Simpson	0.78	0.03	0.80	0.03	94	0.055
	Phylum_Proteobacteria_simpson	0.98	0.01	0.98	0.01	67	0.720
	Phylum_Myxococcota_simpson	0.77	0.24	0.82	0.13	75	0.421
	Phylum_Gemmatimonadota_simpson	0.72	0.23	0.73	0.21	61	0.978
	Phylum_Firmicutes_simpson	0.58	0.41	0.54	0.43	36	0.844
	Phylum_Deinococcota_simpson	0.66	0.15	0.78	0.15	95	**0.048**
	Phylum_Cyanobacteria_simpson	0.81	0.16	0.84	0.15	72	0.524
	Phylum_Chloroflexi_simpson	0.80	0.26	0.82	0.24	55	0.804
	Phylum_Bacteroidota_simpson	0.97	0.01	0.97	0.01	29	0.083
	Phylum_Actinobacteriota_simpson	0.96	0.02	0.96	0.02	56	0.847
	Phylum_Acidobacteriota_simpson	0.88	0.05	0.88	0.06	62	0.934
	Class_Gammaproteobacteria_simpson	0.94	0.03	0.94	0.02	77	0.359
	Class_Bacilli_simpson	0.64	0.42	0.50	0.46	13.5	0.169
	Class_Alphaproteobacteria_simpson	0.97	0.01	0.97	0.01	54	0.762

Supplementary table 4. A) Moisture and dry matter contents of the pooled samples in the intervention (spring n = 3, fall n = 3) and control areas (spring n = 3, fall n = 3). Pairwise permutation results for the B) organic matter and C) moisture content. P-values and q-values are given. Q-values represent p-values after correction for multiple testing using the Benjamini–Hochberg correction.

A) Chemical characteristics of the pooled samples				
Location	Distance	Season	Moisture %	Organic matter %
Intervention area	0	Spring	0.26	1.04
		Fall	0.73	7.16
	5	Spring	0.44	7.40
		Fall	0.71	7.34
	20	Spring	0.35	10.53
		Fall	1.36	18.97
Control area	0	Spring	0.44	6.51
		Fall	0.52	6.30
	5	Spring	0.56	8.90
		Fall	0.32	3.27
	20	Spring	0.44	6.00
		Fall	0.37	4.54

B) Pairwise permutation results for organic matter content			
Comparison	Statistics	*p*	q
Control Fall - Control Spring	-1.558	0.1193	0.4743
Control Fall - Intervention Fall	-1.403	0.1606	0.4743
Control Fall - Intervention Spring	-0.5964	0.5509	0.6611
Control Spring - Intervention Fall	-1.002	0.3162	0.4743
Control Spring - Intervention Spring	0.3072	0.7587	0.7587
Intervention Fall - Intervention Spring	1.005	0.3148	0.4743

C) Pairwise permutation results for moisture content			
Comparison	Statistics	*p*	q
Control Fall - Control Spring	-1.049	0.2943	0.3532
Control Fall - Intervention Fall	-1.715	0.08635	0.1731
Control Fall - Intervention Spring	0.7116	0.4767	0.4767
Control Spring - Intervention Fall	-1.615	0.1064	0.1731
Control Spring - Intervention Spring	1.574	0.1154	0.1731
Intervention Fall - Intervention Spring	1.786	0.07406	0.173

Supplementary Figure 1. Map of Finland showing the locations of the green space gradient experiment (Helsinki, Turku, Lahti, and Arboretum Yltöinen) and the rewilding experiment site in the city of Tampere.

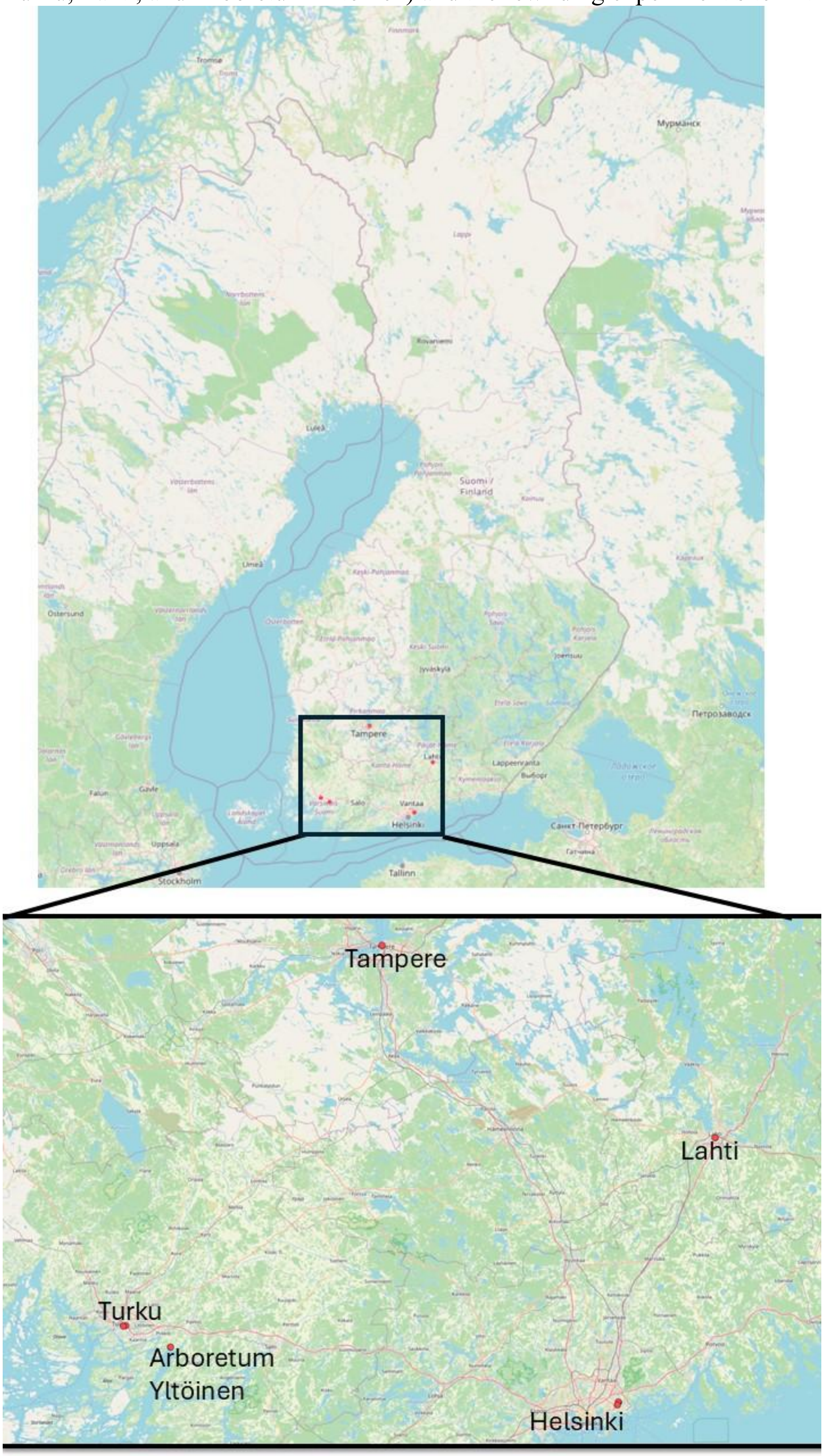

Supplementary Figure 2. Relative abundances (%) of the most common bacterial phyla in the green space gradient experiment with 0, 25, 50, and 100 m distances (n = 5 per distance).

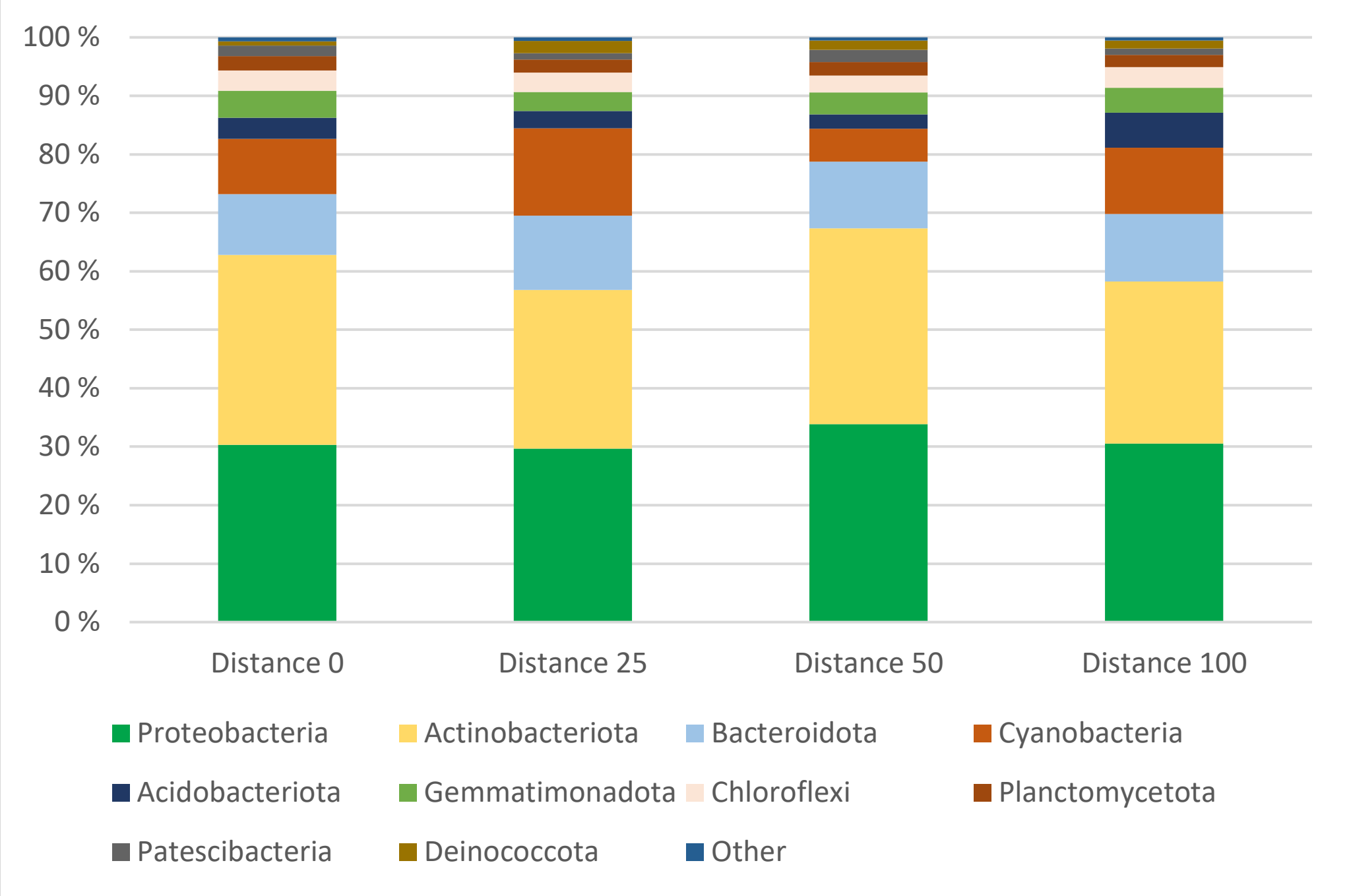

Supplementary Figure 3. Principal Coordinates Analysis (PCoA) of bacterial community compositions at the ASV level based on Aitchison distances. A) Samples grouped by distance from green spaces (0, 25, 50, and 100 m; n = 5 per distance). B) Samples grouped by location (Arboretum Yltöinen n = 4, Helsinki n = 8, Lahti n = 4, and Turku n = 4). Ellipses represent 95% confidence intervals for each group.

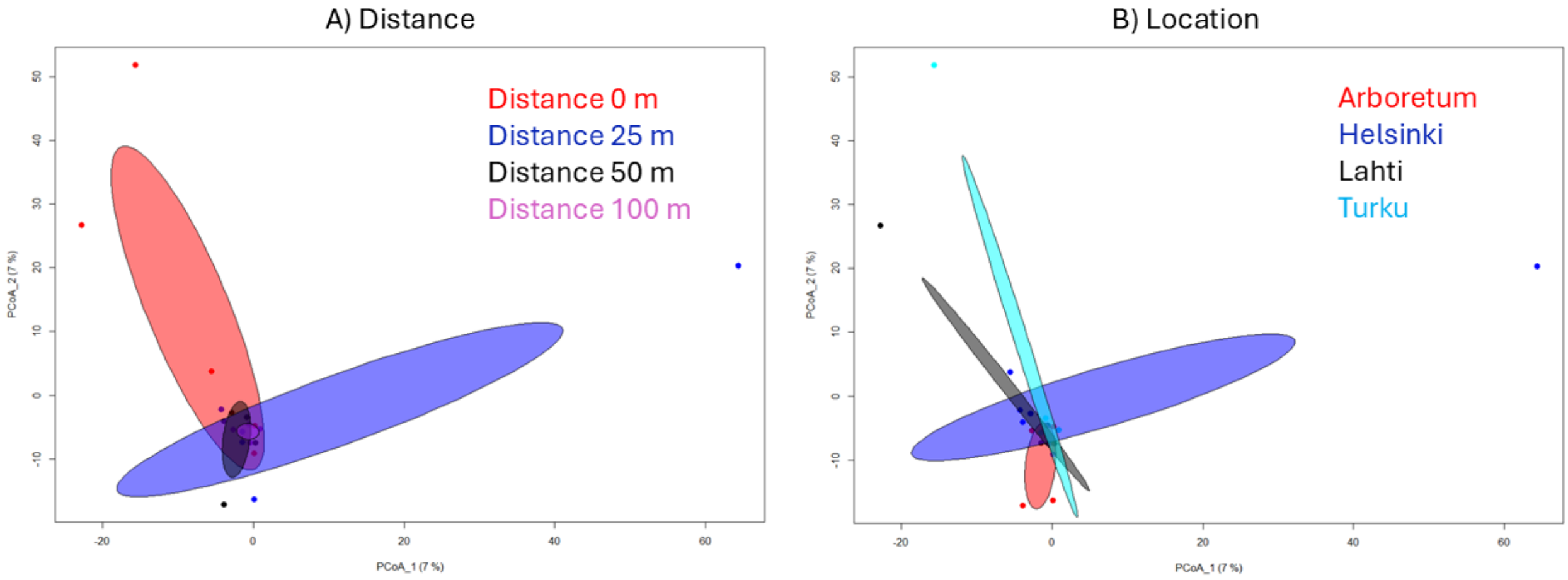

Supplementary Figure 4. Relative abundances (%) of the most common bacterial phyla in the intervention (n = 15 per season, total n = 30) and control area in the rewilding experiment (n = 15 per season, total n =30).

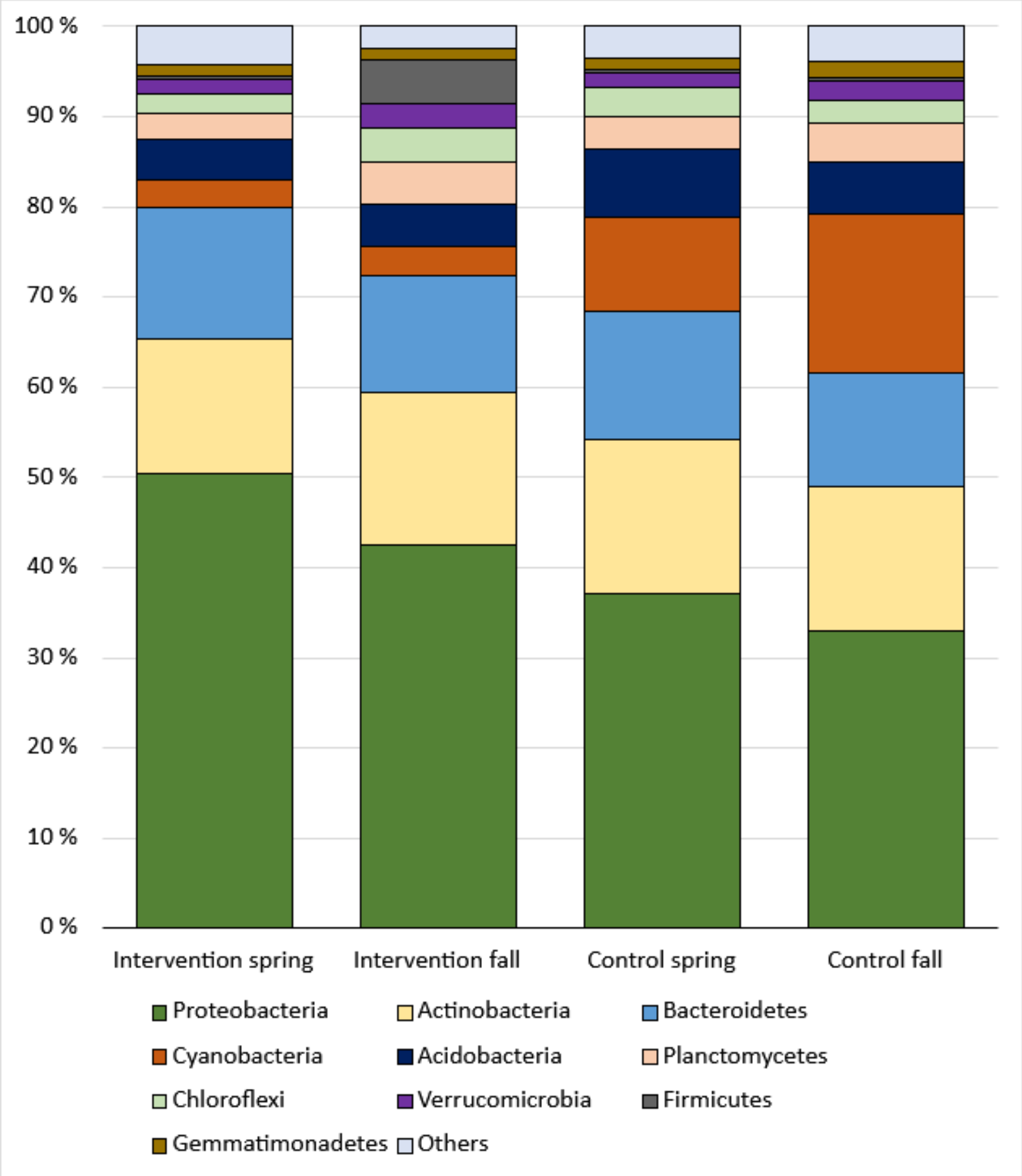

Supplementary figure 5. Principal Coordinates Analysis (PCoA) plot for the rewilding experiment (n=60). PCoA of bacterial community compositions at the genus level based on the Aitchison distances of bacteria in environmental samples collected from the intervention area in the spring (green circles, n = 15) and fall (yellow circles, n = 15) and from the control area in the spring (green triangles, n = 15) and fall (yellow triangles, n = 15) is shown.

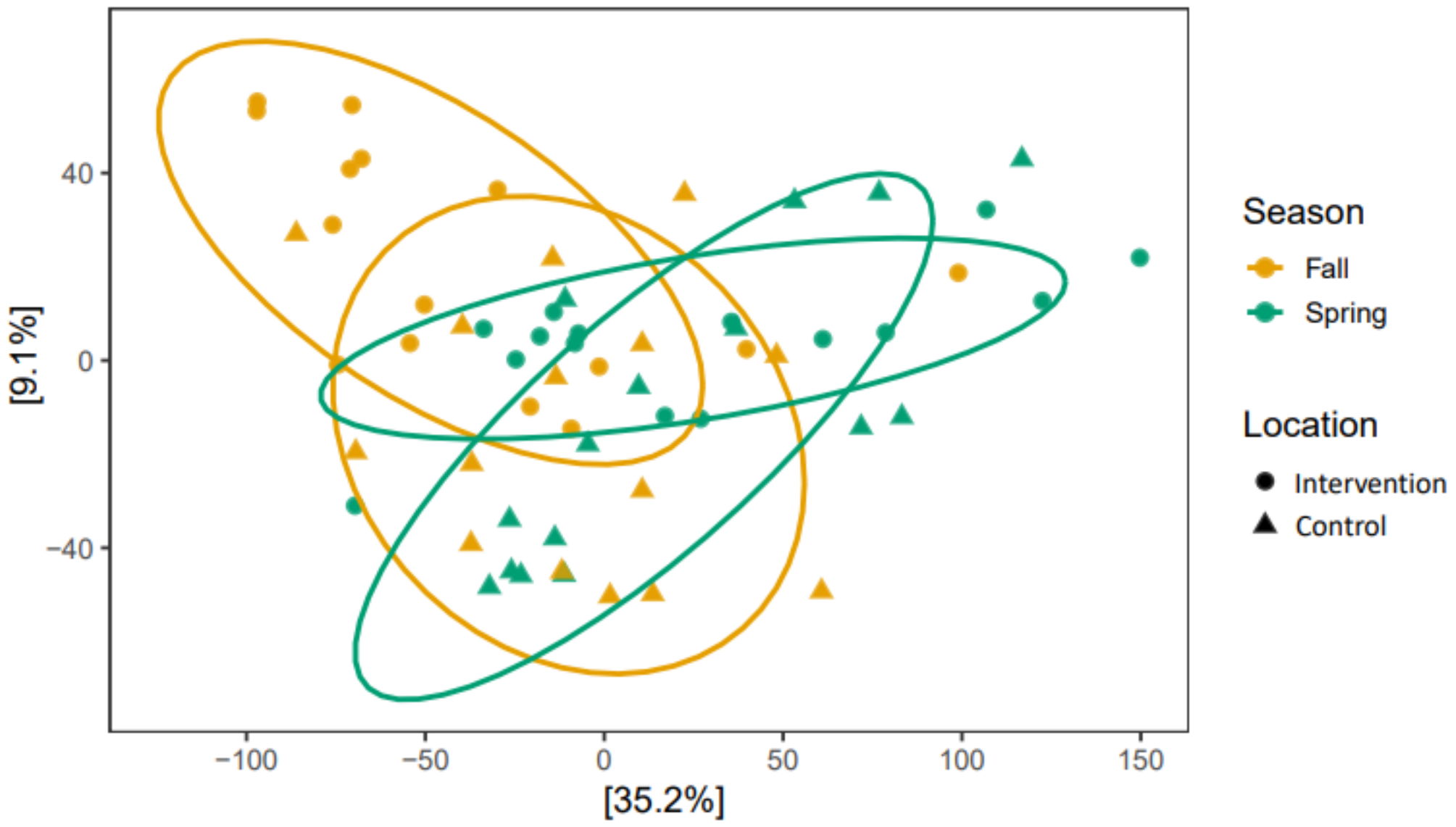

Supplementary Figure 6. Rewilding experiment's intervention area's street paintings (photo by Laura Uimonen).

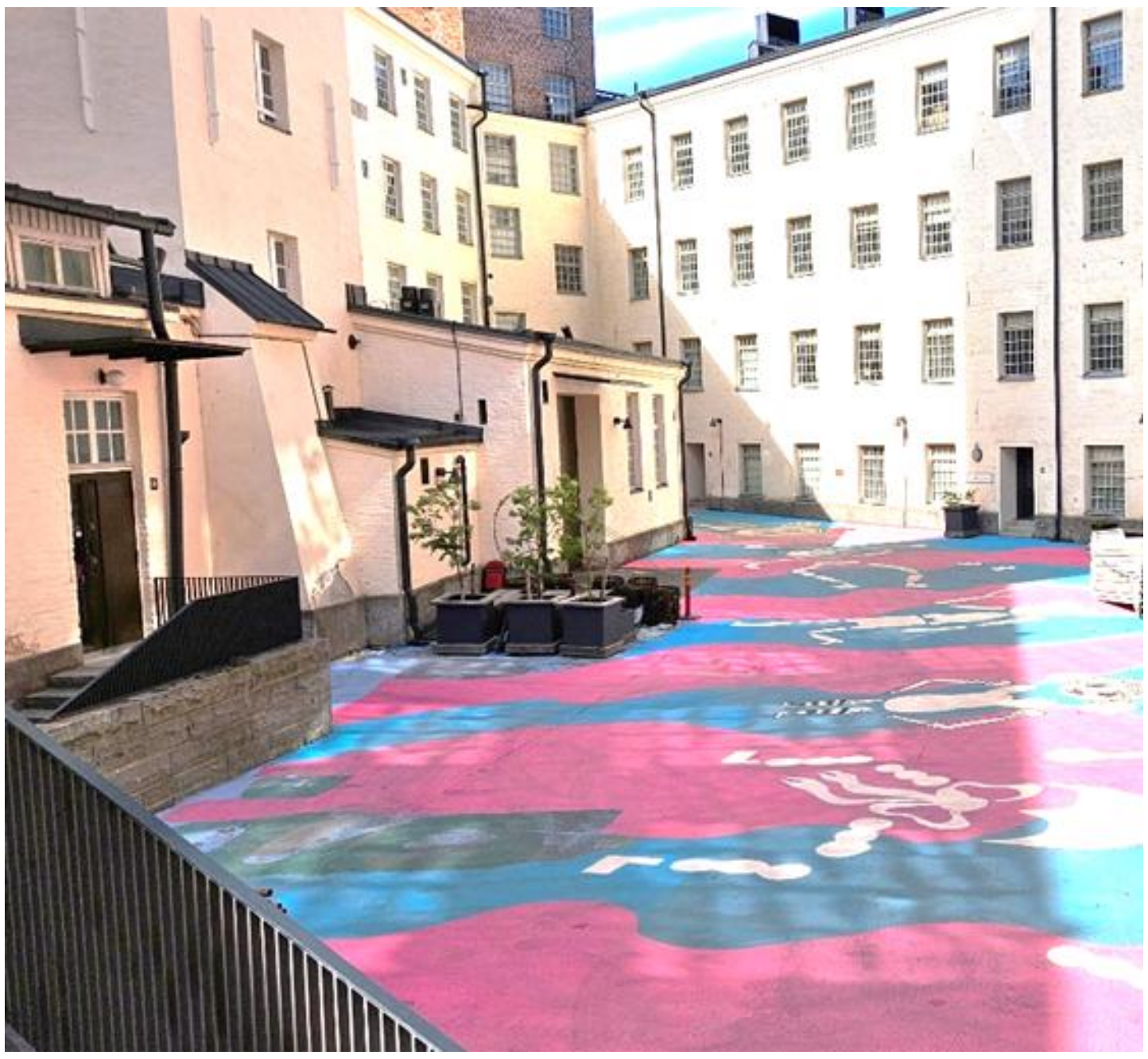

Supplement: Supplementary file 1 [file qvag020_Supplemental_File.pdf]
